# Prevalence and clustering of health behaviours and the association with socio-demographics and mental well-being in Dutch university students

**DOI:** 10.1016/j.pmedr.2023.102307

**Published:** 2023-07-04

**Authors:** Kirsten J.M. van Hooijdonk, Sterre S.H. Simons, Tirza H.J. van Noorden, Sabine A.E. Geurts, Jacqueline M. Vink

**Affiliations:** Behavioural Science Institute, Radboud University, Nijmegen, The Netherlands

**Keywords:** University students, Latent Class Analysis, Health behaviour, Mental well-being, Socio-demographics, Prevention

## Abstract

The college years represent a vulnerable period for developing health-risk behaviours (e.g., physical inactivity/unhealthy eating habits/substance use/problematic internet use/insufficient sleep). This study examined current health behaviour levels (RQ1), health behaviour classes (RQ2) and between-class differences in socio-demographics (RQ3) and mental well-being (RQ4) among Dutch university students (n = 3771). Participants (M_age_ = 22.7 (SD = 4.3); 71.2% female/27.3% male/1.5% other) completed an online survey (Oct-Nov 2021). Descriptive statistics (RQ1), Latent Class Analysis (RQ2), and Kruskal-Wallis/Chi-square tests (RQ3-4) were used. RQ1: Prevalence rates suggest that a subsequent proportion of the student sample engages in health-risk behaviours. RQ2: Four classes were identified: class 1 (n = 862) “Licit substance use health-risk group”, class 2 (n = 435) “Illicit and licit substance use health-risk group”, class 3 (n = 1876) “Health-protective group” and class 4 (n = 598) “Non-substance use health-risk group”. RQ3: Class 1 represents relatively more international students and students in a steady relationship. Class 2 represents relatively more older/male/(pre-)master students and students living with roommates/in a steady relationship/with more financial difficulty. Class 3 represents relatively more younger/female students and students living with family/with lower Body Mass Index (BMI)/less financial difficulty. Class 4 represents relatively more younger/non-Western/international/bachelor students and students living with children/single/part of LGBTIQ+ community/with higher BMI. RQ4: Class 3 has significantly higher mental well-being while class 4 has significantly lower mental well-being, relative to the other classes. Above findings provide new insights which can help educational institutes and governments better understand the clustering of students’ health behaviours and between-class differences in socio-demographics and mental well-being.

## 1 Introduction

Health risk-behaviours^7^ like tobacco smoking, alcohol consumption, unhealthy diet, and physical inactivity have a major impact on people’s risk of developing non-communicable diseases (NCDs) and global mortality rates attributable to these NCDs are high (Spring et al., 2012, World Health Organization., 2018). Especially, combining different health-risk behaviours seems to greatly impact longevity and the more health-risk behaviours are combined the greater the impact (Barbaresko et al., 2018, Khaw et al., 2008, Loef and Walach, 2012, Zhang et al., 2020). Earlier studies among the general population suggested that some health behaviours may be more likely to co-occur with other health behaviours. Clustering seems to exist between: 1) substance use health-risk behaviours, 2) non-substance use health-risk behaviours, 3) combinations of substance/non-substance use health-risk behaviours, and 4) health-protective behaviours (Atorkey et al., 2021, Meader et al., 2016, Noble et al., 2015).

Regarding the initiation and establishment of health-risk behaviours, the college years represent a particularly vulnerable period (Arnett, 2000). Students transit from adolescence to adulthood with new independence and autonomy (Dodd et al., 2021), leading to increases in health-risk behaviours and decreases in health-protective behaviours (Harris et al., 2006, Kwan et al., 2012, Kwan et al., 2013). So far, limited studies have been conducted studying behavioural clusters among students and large differences are observed in study methodologies and included constructs. A deeper understanding of cluster patterns among students is essential as the college years characterize a unique developmental phase and health-risk behaviours established during this period can shape students’ future health and disease prospects (Arnett, 2000). Consequently, providing students with the best possible tools, services, and information to target combined health-risk behaviours during their college years is of great importance to establish healthy lifestyles and reduce future disease burdens. Especially, since already small adaptations in health behavioural patterns can greatly reduce all-cause mortality risks (Barbaresko et al., 2018, Khaw et al., 2008, Loef and Walach, 2012, Zhang et al., 2020).

Next, to understand which groups of students are more likely to present certain patterns of health behaviours, earlier studies have (although limited) studied associations between the clustering of health behaviours and socio-demographics/mental well-being. Among adults, socio-demographics like male gender and caucasian ethnicity have been linked to health-risk behavioural clusters (Atorkey et al., 2021, Meader et al., 2016, Noble et al., 2015), as well as negative mental well-being symptoms like psychological stress, depression and/or anxiety (Bennasar-Veny et al., 2020, Dodd et al., 2010, Ye et al., 2016). However, the generalisability of these findings towards student populations might be limited and the variety in studied socio-demographics and mental well-being measures in relation to health behavioural patterns is restricted.

To bridge these gaps in the literature, the following research questions were addressed. RQ1: What are the current levels of health behaviours? RQ2: Which health behaviour classes can be identified? RQ3: Do the members of the identified health behaviour classes differ in terms of socio-demographics? RQ4: Do the members of the identified health behaviour classes differ in terms of mental well-being? To further extend existing knowledge, a comprehensive set of health behaviours, socio-demographics and mental well-being variables was studied in a large sample of Dutch university students (n = 3771).

## 2 Methods

The study has been pre-registered in the Open Science Framework: https://osf.io/m48xz (van Hooijdonk et al., 2022b).

### 2.1 Procedure and participants

In October-November 2021, all students enrolled at Radboud University^8^ (Nijmegen, The Netherlands) (N = 25035) were invited to complete an online survey of the Healthy Student Life (HSL) project (Healthy Student Life team, 2022). Invitations were sent from the Radboud University mail account to the student’s institutional email addresses. Different recruitment activities were used to increase the response, including sending up to three reminders, distribution of sustainable postcards on campus, and messages on the university’s social media channels and newsletters. Among participants, several prizes were raffled off (vouchers for a weekend trip/online store/campus sports centre). In total 4902 students gave informed consent to participate and request information on their (study) background from the administrative systems (response rate 19.6%). From this sample, data from 3771 students were selected for this paper, representing students who completed the survey section on the health behaviours which were needed to perform the latent class analysis (LCA). The sample in the current study thereby includes 2685 females (71.2%), 1028 males (27.3%) and 58 other gender (1.5%). Mean age was 22.7 years (SD = 4.3). Further socio-demographical characteristics of the sample are represented in Table 1. This study was performed following the Declaration of Helsinki and ethical approval was granted by the Ethics Committee Social Science of Radboud University (ECSW-2021–086).

**Table 1:** Socio-Demographics of Dutch university students (n = 3771; subsample of Healthy Student Life survey Oct-Nov 2021).

Socio-demographics		n (%) / Mean (SD; n)
Age *(Range 17*–*62)*		22.7 (4.3; 3771)
Nationality	Western	3647 (96.7%)
	Non-western	124 (3.3%)
International student	Yes	635 (16.8%)
	No	3101 (82.2%)
Programme level	Bachelor	2111 (56.0%)
	Premaster	245 (6.5%)
	Master	1378 (36.5%)

*Living situation*^a^		
Alone	Yes	507 (13.4%)
	No	3214 (85.2%)
Roommate(s) (no family)	Yes	1705 (45.2%)
	No	2016 (53.5%)
Parent(s)	Yes	1081 (28.7%)
	No	2640 (70.0%)
Partner	Yes	475 (12.6%)
	No	3246 (86.1%)
Child(ren)	Yes	55 (1.5%)
	No	3666 (97.2%)
Other family member(s)	Yes	437 (11.6%)
	No	3284 (87.1%)

Relationship status	Single	1823 (48.3%)
	Married	71 (1.9%)
	Steady relationship	1584 (42.0%)
	Dating	191 (5.1%)
	Other	53 (1.4%)
LGBTIQ+ community	Yes	732 (19.4%)
	No	2988 (79.2%)
Membership student association	Yes	609 (16.1%)
	No	3121 (82.8%)
BMI mean *(Range 11*–*52)*		22.5 (3.5; 3713)
Financial difficulty *(Range 1*–*5)*		2.0 (1.1; 3624)

*Note*. Percentages do not at up to 100% due to missing data.

a Total percentage exceeds 100% as participants could give multiple answers (for example: living with partner and living with children).

### 2.2 Measures

Details on scales and coding are provided in Appendix A.1.

#### 2.2.1 Health behaviours

All available health behaviours (n = 15) were included, including four health behaviours (diet, physical activity, alcohol use and tobacco smoking) which are believed to have the highest contribution to the global disease burden (Atorkey et al., 2021, Meader et al., 2016, Noble et al., 2015). Health behaviours were included in the LCA as categorical variables: (a) Physical activity level: low/moderate/high (International Physical Activity Questionnaire – Short Form (IPAQ-SF) items on vigorous physical activity/moderate physical activity/walking (Craig et al., 2017, IPAQ Research Committee, 2005)). (b) Daily sitting time: 0 to <4/4 to <6/6 to <8/>8 h (IPAQ-SF items on sitting (Craig et al., 2017, IPAQ Research Committee, 2005)). Eating habits: Maximum numbers of days per week students consumed (c) sweet snacks, (d) savoury snacks and (e) fruit and vegetables: range 0–7 days (van den Broek et al., 2020). Licit substance use: (f) Risk of hazardous alcohol use: low-risk (score < 7)/at-risk (score ≥ 7) (Alcohol Use Disorder Identification Test-Consumption (Bush et al., 1998)), use of (g) Tobacco and nicotine ((e-)cigarettes/shag), (h) Cannabis, (i) Hookah: non-user/former user/recent user. Illicit substance use: use of (j) Party stimulant drugs (ecstasy, designer drugs), (k) Other stimulant drugs (amphetamines, cocaine, non-prescribed Ritalin and/or Concerta), (l) Sedative drugs (ketamine, GHB, non-prescribed sleeping and/or sedative drugs): non-user/former user/recent user. (m) Risk of problematic internet use: no-risk (score < 15)/at-risk (score ≥ 15) (Problematic Internet Use Questionnaire Short-Form (Demetrovics et al., 2016)). Sleep (n) duration before a work/study day: ≤4/5/6/7/8/9/≥10 h and (o) quality before a work/study day: very poor/poor/not really good but not bad either/pretty good/very good.

#### 2.2.2 Socio-demographics

Sixteen socio-demographics were included. (a) Gender: male/female/other. (b) Age in years. (c) Nationality: Western/Non-western (Statistics Netherlands, 2022). (d) International student: yes/no. (e) Programme level: bachelor/pre-master/master. Living situation (multiple answers possible) (f) alone, with (g) roommates, (h) parent(s), (i) partner, (j) child(ren), (k) other family: yes/no. (l) Relationship status: single/married/steady relationship/dating/other. (m) Feeling part of the LGBTIQ+ community: yes/no. (n) Membership student association: yes/no. (o) Body Mass Index (BMI). (p) Financial difficulty “I have difficulty paying for things”: 1 = Strongly disagree to 4 = Strongly agree (Watson et al., 2015).

#### 2.2.3 Mental well-being

Nine mental well-being measures were included. (a) Life satisfaction: 1 = Very dissatisfied to 4 = Very satisfied (Cheung and Lucas, 2014). (b) Happiness: 1 = Not happy at all to 10 = Very happy (Abdel-Khalek, 2006). (c) Burnout: mean 12-item Short Burnout Assessment Tool (Schaufeli et al., 2019), items ranged 1 = Never to 5 = Always. (d) Perceived Stress: sum 10-item Perceived Stress Scale (Cohen et al., 1994), items ranged 0 = Never to 4 = Very often. (e) Depression: sum 8-item Center for Epidemiologic Studies – Depression Scale (Van de Velde et al., 2009), items ranged 0 = None or almost none of the time to 3 = All or almost all of the time. (f) Anxiety: sum 2-item Generalized Anxiety Disorder scale (Donker et al., 2011), items ranged 0 = Not at all to 3 = Nearly every day. (g) Study engagement: mean 9-item shortened student version of the Utrecht Work Engagement Scale (Schaufeli and Bakker, 2004), items ranged 0 = Never to 6 = Always/Every day. Statement COVID-19 concerns regarding the impact on (h) social life and contacts and (i) future prospects job market: 1 = Strongly disagree to 5 = Strongly agree.

### 2.3 Statistical analyses

Analyses were performed in SPSS version 25 and R studio (IBM Corp, 2017, RStudio Team, 2020). First, descriptives were run to investigate participants’ socio-demographics (descriptive statistics), health behaviour (RQ1) and mental well-being prevalence rates (including interpretation of scores, see Appendix A.2). Second, health behavioural patterns (RQ2) were investigated using LCA (poLCA package in R (Linzer and Lewis, 2011)) including 15 categorical health behaviours. LCA can be used to identify unobserved subgroups based on observed variables and results in probability scores (between 0 and 1) per response category and identified class (Nylund-Gibson and Choi, 2018, Vermunt and Magidson, 2002). Missing data (assumed Missing at Random) were handled within the Expectation-Maximization algorithm (Nylund-Gibson and Choi, 2018). The optimal amount of classes was determined by first fitting a one-class model and then increasing the amount of classes by one, up to a six-class model (Nylund-Gibson and Choi, 2018). To avoid local solutions, 300 iterations of each model were run using random starting values. The model that best fitted the data was selected based on the lowest Bayesian information criterion (BIC) and Akaike information criterion (AIC) score (Linzer and Lewis, 2011). In case the lowest BIC and AIC values pointed to different best-fitting models, the lowest BIC value was decisive (Linzer and Lewis, 2011, Weller et al., 2020). After class determination, a new variable “class membership” was constructed (by using “predclass”) allocating all participants to one of the classes. For the best model, all probabilities per health behaviour response category and class were compared and descriptive labels were added. Third, between-class differences in socio-demographics (RQ3) and mental well-being (RQ4) were investigated using Kruskal-Wallis tests for continuous variables (due to violation of normal distribution (all variables) and homogeneity of variances assumptions (most variables)) and Chi-square tests for categorical variables. To correct for multiple testing, Bonferroni correction was used to adjust the critical *p*-values for the between-class tests (adjusted *p*-value socio-demographics 0.003; 0.05/16 variables and adjusted *p*-value mental well-being 0.006; 0.05/9 variables). Post hoc tests were performed when between-class differences were identified (continuous variables: Mann-Whitney *U* test, categorical variables: Chi-square test). Again, Bonferroni correction was used to correct for multiple testing (adjusted *p*-value 0.008; 0.05/6 comparisons).

## 3 Results

### 3.1 Descriptive statistics

Mean age was 22.7 years (Table 1). Most students were female (71.2%), had a Western nationality (96.7%), were non-international (82.2%), followed a bachelor programme (56.0%) and were not living alone (85.2%). Additionally, 48.3% were single, 19.4% felt part of the LGBTIQ+ community, and 16.1% was a student association member. The mean BMI was 22.5 and the mean financial difficulty was 2.0 (on a scale from 1 = low to 5 = high).

### 3.2 Prevalence of health behaviours (RQ1)

Almost 9 out of 10 students (87.5%) have a moderate to high physical activity level and almost 2 out of 3 students (61.7%) sit ≥8 h per day (Table 2). On average, students consume sweet snacks, savoury snacks and fruit/vegetables on respectively 3, 2 and 6 days/week. Almost 1 out of 10 students (8.5%) is at-risk for hazardous alcohol use and 17.4% has smoked (e-)cigarettes/shag in the past half-year. Recent cannabis use was reported by 24.1% of the students while 4.2% of the students reported recent hookah use. The prevalence of recent party stimulant drugs was the highest (11.4%) compared to other stimulant (5.4%) and sedative substances (5.6%). Over 1 out of 4 students (28.7%) is at-risk for problematic internet use. Last, sleeping <7 h/night before a work/study day was reported by 18.5% and a (very) poor sleep quality was reported by 15.9%.

**Table 2:** Prevalence of Health Behaviours among Dutch university students (n = 3771; subsample of Healthy Student Life survey Oct–Nov 2021).

Health behaviour		n (%) / Mean (SD; n)
Physical activity level	Low	359 (9.5%)
	Moderate	1571 (41.7%)
	High	1728 (45.8%)
Daily sitting time	0 to <4 h	120 (3.2%)
	4 to <6 h	420 (11.1%)
	6 to <8 h	848 (22.5%)
	≥8 h	2325 (61.7%)

*Eating habits*		
Sweet snacks	Max days per week	2.6 (1.8; 3770)
Savoury snacks	Max days per week	1.9 (1.3; 3770)
Fruit and vegetables	Max days per week	6.0 (1.3; 3770)

*Licit substance use*		
Hazardous alcohol use	Low-risk (AUDIT-C score < 7)	3438 (91.2%)
	At-risk (AUDIT-C score ≥ 7)	320 (8.5%)
Tobacco/nicotine	Non-user	2932 (77.8%)
	Former user	181 (4.8%)
	Recent user	658 (17.4%)
Cannabis	Non-user	2130 (56.5%)
	Former user	730 (19.4%)
	Recent user	907 (24.1%)
Hookah	Non-user	2973 (78.8%)
	Former user	637 (16.9%)
	Recent user	159 (4.2%)

*Illicit substance use*		
Party stimulant drugs	Non-user	3012 (79.9%)
	Former user	327 (8.7%)
	Recent user	429 (11.4%)
Other stimulant substances	Non-user	3296 (87.4%)
	Former user	270 (7.2%)
	Recent user	202 (5.4%)
Sedative substances	Non-user	3305 (87.6%)
	Former user	251 (6.7%)
	Recent user	212 (5.6%)

Problematic internet use	No-risk (Problematic internet score < 15)	2688 (71.3%)
	At-risk (Problematic internet score ≥ 15)	1083 (28.7%)

*Sleep*		
Duration	≤4 h	30 (0.8%)
	5 h	129 (3.4%)
	6 h	540 (14.3%)
	7 h	1451 (38.5%)
	8 h	1343 (35.6%)
	9 h	245 (6.5%)
	≥10 h	33 (0.9%)
Quality	Very poor	101 (2.7%)
	Poor	497 (13.2%)
	Not really good but not bad either	1261 (33.4%)
	Pretty good	1594 (42.3%)
	Very good	318 (8.4%)

*Note*. Percentages do not at up to 100% due to missing data.

### 3.3 Latent class analysis of health behaviours (RQ2)

Based on the lowest BIC value, the best model included 4 classes (Table 3).

**Table 3:** Model Fit Statistics for each Fitted Latent Class Model of Health Behaviours among Dutch university students (n = 3771; subsample of Healthy Student Life survey Oct-Nov 2021).

Class	BIC	AIC
1	106427.4	106115.6
2	102444.9	101815.1
3	102036.7	101089.5
**4**	**101917.5**	**100651.7**
5	102047.8	100464.1
6	102266.7	100365.0

*Note*. AIC = Akaike information criterion; BIC = Bayesian information criterion. Best model presented in bold.

**Fig. 1:**
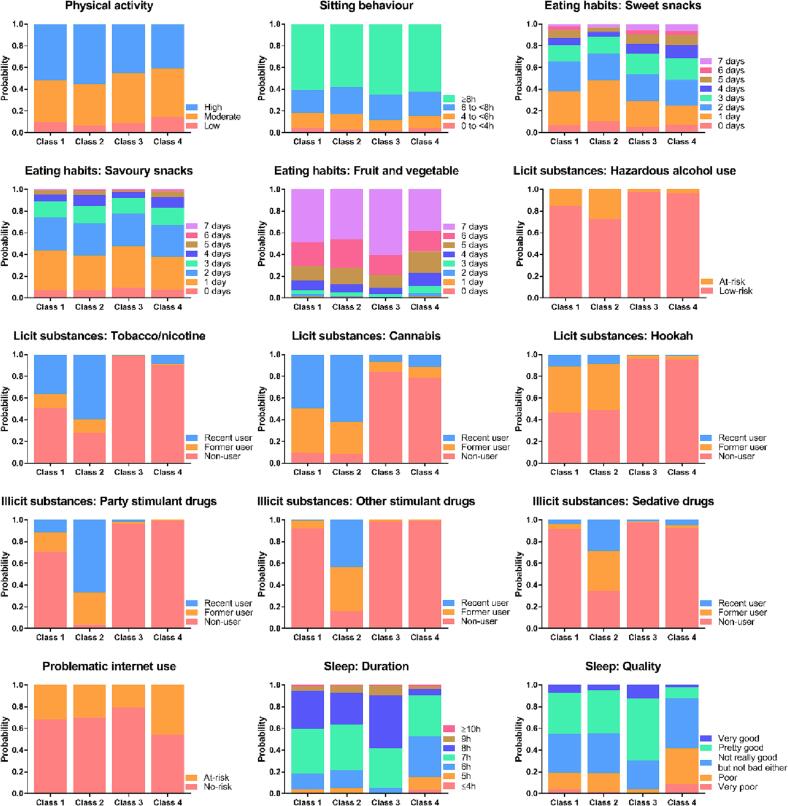
Probabilities per health behaviour, resulting from the Latent Class Analysis (LCA) performed among Dutch university students (n = 3771; subsample of Healthy Student Life survey Oct-Nov 2021). *Note*. Probabilities per health behaviour and per response category are presented for all 15 health behaviours included in the LCA. Class 1 (n = 862): “Licit substance use health-risk group”. Class 2 (n = 435): “Illicit and licit substance use health-risk group”. Class 3 (n = 1876): “Health-protective group”. Class 4 (n = 598): “Non-substance use health-risk group”.

All probabilities have been visually presented in Fig. 1 and added to Appendix Table A.3. These probabilities per response category suggest how likely a person in a class will present certain health-risk or protective behaviour. E.g., the probability of recent cannabis use is 0.50 for class 1, 0.62 for class 2, 0.07 for class 3 and 0.11 for class 4, suggesting that class 2 has the highest probability for recent cannabis use, followed by class 1 while both class 3 and 4 present lowest probabilities for recent cannabis use. Based on these observed patterns and most remarkable characteristics, descriptive labels were added:

*Class 1 (n = 862):* “*Licit substance use health-risk group*”. This class (and class 2) has high probabilities for former/recent use of cannabis, tobacco/nicotine and hookah compared to the other classes. Additionally, this class represents students with higher probabilities for hazardous alcohol use than class 3 and 4, but lower than class 2. Last, this class has low probabilities for former/recent use of illicit substances (party drugs/stimulant drugs/sedative drugs).

*Class 2 (n = 435):* “*Illicit and licit substance use health-risk group*”. This class represents students with highest probabilities of hazardous alcohol use and highest probabilities of recent use of most (il)licit substances compared to the other classes. In contrast, this class also represents students with highest probabilities of having a high physical activity level and sitting <8 h/day (although in general all classes had relatively high probabilities to sit ≥8 h/day).

*Class 3 (n = 1876):* “*Health-protective group*”. Compared to the other classes, this class represents students with highest probabilities to eat fruit/vegetables daily, to sleep ≥7 h/night and to have good quality sleep. Additionally, low probabilities for hazardous alcohol use, (il)licit substance use and problematic internet use were observed. In contrast, class 3 also represent students with highest probabilities to sit ≥8 h/day.

*Class 4 (n = 598):* “*Non-substance use health-risk group*”. This class represents students with highest probability for problematic internet use compared to the other classes. Additionally, students in this class have highest probabilities to have a low/moderate physical activity level, to not daily consume fruit/vegetables, to have insufficient sleep (<7 h/night) as well as to have (very) poor sleep quality. Last, students in this class have lower probabilities for hazardous alcohol use and (il)licit substance use.

### 3.4 Between-class differences in socio-demographics (RQ3)

Differences between (some of) the four classes were found for all socio-demographics, except living situation categories alone and with partner and membership student association (Table 4). Class 1 represents relatively more international students (together with class 4) and students in a steady relationship (not statistically significant for class 1 vs. 2).

**Table 4:** Between-Class Differences in Socio-Demographics among Dutch university students (n = 3771; subsample of Healthy Student Life survey Oct-Nov 2021).^a^

Socio-demographics	Class						*p*-value KW / X^2^ test	Post hoc test		
								Class 1 vs.	Class 2 vs.	Class 3 vs.
Age		Mean (SD; n)								
	1	22.8 (3.3; 862)					**<0.001**	Ref	–	–
	2	23.6 (2.9; 435)						**<0.001**	Ref	–
	3	22.5 (4.8; 1876)						**<0.001**	**<0.001**	Ref
	4	22.5 (4.4; 598)						**<0.001**	**<0.001**	0.679
Gender		Male (n (%))	Female (n (%))	Other (n (%))						
	1	279 (32.4%)	569 (66.0%)	14 (1.6%)			**<0.001**	Ref	–	–
	2	178 (40.9%)	249 (57.2%)	8 (1.8%)				0.008	Ref	–
	3	408 (21.7%)	1447 (77.1%)	21 (1.1%)				**<0.001**	**<0.001**	Ref
	4	163 (27.3%)	420 (70.2%)	15 (2.5%)				0.068	**<0.001**	**<0.001**
Nationality		Western (n (%))	Non-western (n (%))							
	1	838 (97.2%)	24 (2.8%)				**<0.001**	Ref	–	–
	2	429 (98.6%)	6 (1.4%)					0.112	Ref	–
	3	1822 (97.1%)	54 (2.9%)					0.891	0.076	Ref
	4	558 (93.3%)	40 (6.7%)					**<0.001**	**<0.001**	**<0.001**
International student		Yes (n (%))	No (n (%))							
	1	222 (26.3%)	623 (73.7%)				**<0.001**	Ref	–	–
	2	53 (12.3%)	379 (87.7%)					**<0.001**	Ref	–
	3	225 (12.1%)	1642 (87.9%)					**<0.001**	0.901	Ref
	4	135 (22.8%)	457 (77.2%)					0.134	**<0.001**	**<0.001**
Programme level		Bachelor (n (%))	Master (n (%))	Premaster (n (%))						
	1	471 (55.8%)	311 (36.8%)	62 (7.3%)			**<0.001**	Ref	–	–
	2	201 (46.5%)	186 (43.1%)	45 (10.4%)				**0.005**	Ref	–
	3	1056 (56.6%)	699 (37.5%)	111 (5.9%)				0.387	**<0.001**	Ref
	4	383 (64.7%)	182 (30.7%)	27 (4.6%)				**0.002**	**<0.001**	**0.002**
*Living situation*		Yes (n (%))	No (n (%))							
Alone	1	110 (13.0%)	737 (87.0%)				0.357			
	2	63 (14.8%)	363 (85.2%)							
	3	242 (13.0%)	1615 (87.0%)							
	4	92 (15.6%)	499 (84.4%)							
Roommate(s) (no family)	1	471 (55.6%)	376 (44.4%)				**<0.001**	Ref	–	–
	2	253 (59.4%)	173 (40.6%)					0.199	Ref	–
	3	747 (40.2%)	1110 (59.8%)					**<0.001**	**<0.001**	Ref
	4	234 (39.6%)	357 (60.4%)					**<0.001**	**<0.001**	0.785
Parent(s)	1	160 (18.9%)	687 (81.1%)				**<0.001**	Ref	–	–
	2	54 (12.7%)	372 (87.3%)					**0.005**	Ref	–
	3	665 (35.8%)	1192 (64.2%)					**<0.001**	**<0.001**	Ref
	4	202 (34.2%)	389 (65.8%)					**<0.001**	**<0.001**	0.470
Partner	1	119 (14.0%)	728 (86.0%)				0.312			
	2	58 (13.6%)	368 (86.4%)							
	3	234 (12.6%)	1623 (87.4%)							
	4	64 (10.8%)	527 (89.2%)							
Child(ren)	1	3 (0.4%)	844 (99.6%)				**<0.001**	Ref	–	–
	2	5 (1.2%)	421 (98.8%)					0.081	Ref	–
	3	29 (1.6%)	1828 (98.4%)					**0.007**	0.551	Ref
	4	18 (3.0%)	573 (97.0%)					**<0.001**	0.048	0.022
Other family member(s)	1	55 (6.5%)	792 (93.5%)				**<0.001**	Ref	–	–
	2	23 (5.4%)	403 (94.6%)					0.442	Ref	–
	3	271 (14.6%)	1586 (85.4%)					**<0.001**	**<0.001**	Ref
	4	88 (14.9%)	503 (85.1%)					**<0.001**	**<0.001**	0.859
Relationship status		Single (n (%))	Married (n (%))	Steady relationship (n (%))	Dating (n (%))	Other (n (%))				
	1	336 (39.7%)	13 (1.5%)	410 (48.4%)	70 (8.3%)	18 (2.1%)	**<0.001**	Ref	–	–
	2	169 (39.7%)	5 (1.2%)	205 (48.1%)	32 (7.5%)	15 (3.5%)		0.627	Ref	–
	3	980 (52.7%)	36 (1.9%)	772 (41.6%)	58 (3.1%)	12 (0.6%)		**<0.001**	**<0.001**	Ref
	4	338 (57.2%)	17 (2.9%)	197 (33.3%)	31 (5.2%)	8 (1.4%)		**<0.001**	**<0.001**	**<0.001**
LGBTIQ+ community		Yes (n (%))	No (n (%))							
	1	192 (22.7%)	655 (77.3%)				**<0.001**	Ref	–	–
	2	81 (19.0%)	345 (81.0%)					0.134	Ref	–
	3	299 (16.1%)	1557 (83.9%)					**<0.001**	0.147	Ref
	4	160 (27.1%)	431 (72.9%)					0.056	**0.003**	**<0.001**
Membership student association		Yes (n (%))	No (n (%))							
	1	145 (17.1%)	705 (82.9%)				0.011			
	2	83 (19.4%)	344 (80.6%)							
	3	309 (16.6%)	1551 (83.4%)							
	4	72 (12.1%)	521 (87.9%)							
BMI Mean		Mean (SD; n)								
	1	22.8 (3.6; 852)					**<0.001**	Ref	–	–
	2	22.6 (3.0; 431)						0.821	Ref	–
	3	22.2 (3.3; 1841)						**<0.001**	**<0.001**	Ref
	4	23.0 (4.4; 589)						0.852	0.914	**<0.001**
Financial difficulty		Mean (SD; n)								
	1	2.1 (1.2; 824)					**<0.001**	Ref	–	–
	2	2.4 (1.2; 409)						**<0.001**	Ref	–
	3	1.8 (1.0; 1812)						**<0.001**	**<0.001**	Ref
	4	2.2 (1.2; 579)						0.262	0.030	**<0.001**

*Note*. KW = Kruskal-Wallis test; X^2^ = Chi-square test. Post hoc tests: Mann-Whitney *U* (continuous variables), Chi-square (categorical variables). Class 1 (n = 862): “Licit substance use health-risk group”. Class 2 (n = 435): “Illicit and licit substance use health-risk group”. Class 3 (n = 1876): “Health-protective group”. Class 4 (n = 598): “Non-substance use health-risk group”.

a Bonferroni correction: *P* < 0.003 was deemed statistically significant (0.05/16 variables) for the between-class analyses. *P* < 0.008 was deemed statistically significant (0.05/6 comparisons) for the post hoc tests. Statistically significant results are presented in bold.

The students representing class 2 are significantly older and relatively more often male (not statistically significant for class 1 vs. 2). Additionally, class 2 represents relatively more students enrolled in (pre-)master programmes, students living with roommates (not statistically significant for class 1 vs. 2), students in a steady relationship (not statistically significant for class 1 vs. 2) and students experiencing significantly higher levels of financial difficulty (not statistically significant for class 2 vs. 4).

Class 3 represents significantly younger students (together with class 4), relatively more females, students living with parents/other family (not statistically different for class 3 vs. 4) and students with lowest mean BMI and financial difficulty levels compared to the other classes.

Class 4 represents relatively more younger students (together with class 3), students with a non-western nationality, international students (together with class 1) and bachelor students. Additionally, relatively more students who live with children (only statistically significant for class 1 vs. 4) and single students are present. Last, class 4 represent relatively more students who feel part of the LGBTIQ+ community (not statistically different for class 1 vs. 4) and students with higher mean BMI levels (only significantly different for class 1 vs. 4).

### 3.5 Between-class differences in mental well-being (RQ4)

The four classes differed significantly (*p* < 0.001) on all mental well-being measures (Table 5). Post hoc test indicated that students representing class 3 have significantly higher levels of mental well-being than class 1, 2 (except COVID-19 concerns regarding social life and contacts) and 4. Students representing class 4 have significantly lower levels of mental well-being than class 1 and 2 (except study engagement and COVID-19 concerns). No between-class differences were identified between class 1 and 2 (except for study engagement).

**Table 5:** Between-Class Differences in Mental Well-being among Dutch university students (n = 3771; subsample of Healthy Student Life survey Oct-Nov 2021).^a^

Mental well-being	Class	Mean (SD; n)	*p*-value KW test	Post hoc test		
				Class 1 vs.	Class 2 vs.	Class 3 vs.
Life satisfaction	1	3.0 (0.6; 862)	**<0.001**	Ref	–	–
	2	3.0 (0.6; 435)		0.831	Ref	–
	3	3.1 (0.6; 1875)		**<0.001**	**<0.001**	Ref
	4	2.8 (0.6; 598)		**<0.001**	**<0.001**	**<0.001**
Happiness	1	6.8 (1.5; 862)	**<0.001**	Ref	–	–
	2	6.7 (1.5; 435)		0.612	Ref	–
	3	7.2 (1.3; 1875)		**<0.001**	**<0.001**	Ref
	4	6.2 (1.6; 598)		**<0.001**	**<0.001**	**<0.001**
Burnout (mean)	1	2.8 (0.7; 862)	**<0.001**	Ref	–	–
	2	2.9 (0.7; 435)		0.035	Ref	–
	3	2.5 (0.6; 1875)		**<0.001**	**<0.001**	Ref
	4	3.0 (0.6; 598)		**<0.001**	**<0.001**	**<0.001**
Perceived stress (sum)	1	17.8 (7.0; 862)	**<0.001**	Ref	–	–
	2	17.6 (7.0; 435)		0.506	Ref	–
	3	15.3 (6.4; 1875)		**<0.001**	**<0.001**	Ref
	4	20.3 (6.6; 598)		**<0.001**	**<0.001**	**<0.001**
Depression (sum)	1	8.9 (4.6; 862)	**<0.001**	Ref	–	–
	2	9.1 (4.2; 435)		0.281	Ref	–
	3	7.3 (4.0; 1876)		**<0.001**	**<0.001**	Ref
	4	11.2 (4.6; 598)		**<0.001**	**<0.001**	**<0.001**
Anxiety (sum)	1	2.5 (1.8; 862)	**<0.001**	Ref	–	–
	2	2.4 (1.7; 435)		0.427	Ref	–
	3	1.9 (1.5; 1875)		**<0.001**	**<0.001**	Ref
	4	3.0 (1.8; 598)		**<0.001**	**<0.001**	**<0.001**
Study engagement (mean)	1	3.4 (1.1; 862)	**<0.001**	Ref	–	–
	2	3.2 (1.2; 435)		**0.005**	Ref	–
	3	3.7 (1.1; 1876)		**<0.001**	**<0.001**	Ref
	4	3.3 (1.1; 598)		0.068	0.285	**<0.001**

*COVID-19 concerns*						
Social life and contacts	1	3.7 (1.2; 821)	**<0.001**	Ref	–	–
	2	3.6 (1.3; 412)		0.233	Ref	–
	3	3.4 (1.2; 1815)		**<0.001**	0.009	Ref
	4	3.7 (1.2; 578)		0.739	0.402	**<0.001**
My future prospects in the job market	1	3.0 (1.2; 821)	**<0.001**	Ref	–	–
	2	2.9 (1.2; 412)		0.213	Ref	–
	3	2.7 (1.2; 1816)		**<0.001**	**0.006**	Ref
	4	3.0 (1.3; 578)		0.790	0.184	**<0.001**

*Note*. KW = Kruskal-Wallis test. Post hoc tests: Mann-Whitney *U*. Class 1 (n = 862): “Licit substance use health-risk group”. Class 2 (n = 435): “Illicit and licit substance use health-risk group”. Class 3 (n = 1876): “Health-protective group”. Class 4 (n = 598): “Non-substance use health-risk group”.

a Bonferroni correction: *P* < 0.006 was deemed statistically significant (0.05/9 variables) for the between-class analyses. *P* < 0.008 was deemed statistically significant (0.05/6 comparisons) for the post hoc tests. Statistically significant results are presented in bold.

## 4 Discussion

### 4.1 Principal findings

This study among Dutch university students indicated that: (RQ1) a subsequent proportion of the students engaged in health-risk behaviours, (RQ2) four distinct classes of health behaviours were found, between-class differences were present for (RQ3) most studied socio-demographics and (RQ4) all investigated mental well-being measures.

Regarding RQ1, overall, students highly engage in health-risk behaviours. This is largely in line with prevalence’s mentioned in previous studies (Busse et al., 2021, Hutchesson et al., 2021, Quick et al., 2014, van Hooijdonk et al., 2022a).

Regarding RQ2, we identified four health behavioural classes. The identification of the substance use high-risk classes (class 1 licit and 2 (il)licit substances) aligns with previous studies involving Iranian, American and Canadian students who also identified a class of (il)licit substances (Kwan et al., 2016, Luo et al., 2015, Mohammadpoorasl et al., 2013). The identification of class 3 (“Health-protective group”) and class 4 (“Non-substance use health-risk group”) also aligns with previous studies as at least one class presenting health-protective behaviours and one class presenting non-substance use health-risk behaviours were identified (Bennasar-Veny et al., 2020, Laska et al., 2009, Luo et al., 2015).

Regarding RQ3, our findings imply that certain groups of students are more likely to express certain patterns of health-risk or health-protective behaviours. Class 1 (“Licit substance use health-risk group”) consisted of relatively more international students. This aligns with a previous study, where we saw that students not born (vs. born) in the Netherlands were more likely to use tobacco/cannabis on a weekly basis (van Hooijdonk et al., 2022a). Future studies should explore whether substance use was initiated before or after arrival in the Netherlands. In some countries, tobacco smoking and alcohol use prevalence is higher compared to the Netherlands (Eurostat, 2019a, Eurostat, 2019b), which might explain why international students were relatively more often present in class 1. Alternatively, international students could have initiated or increased their substance use in the Netherlands to cope with new challenges or due to a more liberal and tolerant Dutch substance use policy (Garretsen, 2010). Class 2 (“Illicit and licit substance use health-risk group”) consisted of relatively more students who were older, male and living with roommates. This aligns with previous studies that showed that youth start experimenting with licit (vs. illicit) substances at an earlier age (Alcover and Thompson, 2020). Additionally, males (vs. females) are more often engaged in illicit substance use (McHugh et al., 2018) and not living with parents facilitates substance use (van Hooijdonk et al., 2022a). Class 3 (“Health-protective group”) was more often represented by students living with parents and experiencing less financial difficulty. Dutch policy (student grant system was replaced by a student loan system) and shortage in student housing might have influenced this, as more students postponed moving out of their parental homes to avoid financial problems (ABF Research, 2021). This implies that living with parents serves as a protective factor in students’ substance use (Boot et al., 2009) and other health-risk behaviours. Class 4 (“Non-substance use health-risk group”) consisted of relatively more non-western/international students and students who feel part of the LGBTIQ+ community. Previous studies suggest that the likelihood of problematic internet use is associated with decreased offline social support (Mazzoni et al., 2016, Ramón-Arbués et al., 2021). Although speculative, it might be that non-western/international students/students who feel part of the LGBTIQ+ community have experienced less offline social support (possibly due to the COVID-19 pandemic or due to stigma towards the LGBTIQ+ community (Johns et al., 2019)), therefore impacting their internet use and possibly also other health-risk behaviours.

Regarding RQ4, the classes differed on all measured mental well-being variables. Aligning with previous studies (Bennasar-Veny et al., 2020, Dodd et al., 2010, Kwan et al., 2016, Ye et al., 2016), class 3 (“Health-protective group”) scored best on all mental well-being measures, while class 4 (“Non-substance use health-risk group”) scored significantly lower on most mental well-being measures compared to the other classes. This suggests a positive relation between combinations of health-protective behaviours and mental well-being, as well as a negative relation between combinations of certain health-risk behaviours and mental well-being. As a cross-sectional design was used, the direction of these relationships cannot be determined. However, previous studies (on specific health-risk behaviours instead of clusters) showed that these relationships could be bi-directional (Alvaro et al., 2013, Azevedo Da Silva et al., 2012, Polivy and Herman, 2005, Treur et al., 2021, Yang et al., 2022), or underlying factors (like genetic susceptibility) could explain co-occurrence (Abdellaoui et al., 2021, Vink and Schellekens, 2018). Last, our study included both negative (often reported) and positive (often lacking) mental well-being measures which were all consistently linked to the identified classes.

### 4.2 Implications

This study enhances the global understanding of complex health behavioural patterns. As college students are highly susceptible to health-risk behaviours, already small lifestyle changes can greatly impact future health/disease prospects (Barbaresko et al., 2018, Khaw et al., 2008, Loef and Walach, 2012, Zhang et al., 2020). Therefore, supporting and educating students on health behaviours is essential for establishing lifelong healthy habits and governments/higher educational institutes play an important role in providing adequate, low-threshold and tailored services/interventions. Understanding the clustering of health behaviours and associations with socio-demographics/well-being variables can shape the development of tailored interventions/services which match the needs/characteristics of different student groups and possibly target health behavioural combinations that may otherwise have remained overlooked and ignored.

### 4.3 Strengths, limitations and future research

Our study has several strengths. (1) The used LCA is a data-driven approach with multiple advantages compared to standard cluster approaches (Vermunt and Magidson, 2002). (2) We included a broad perspective with a comprehensive set of health behaviours, socio-demographics and mental well-being variables. This enabled us to make more detailed descriptive profiles of which students engage in health-risk or protective behaviours and are more likely to have lower/higher mental well-being.

However, several limitations need to be acknowledged. First, a response bias might exist as 19.6% of all students joined the study. Possibly, these students are not completely representative of all students. Second, the gender distribution (71.2% female/27.3% male) was different from the biological sex distribution of the total student population (58% female/42% male; internal source). This might impacted the composition of the classes and associations with socio-demographics/mental well-being. Next, we used self-report measures possibly resulting in underreporting of health-risk behaviours and mental well-being symptoms due to social desirability (Tourangeau and Yan, 2007). Furthermore, data were collected in fall 2021 when still several restrictions as a consequence of the COVID-19 pandemic were in place (Rijksinstituut voor Volksgezondheid en Milieu, 2022), which possibly impacted our findings. Finally, our correlational design prohibits causal conclusions and our study is limited to a specific student population and location which could potentially reduce the generalizability of our findings to other (international) student populations. However, as reflected by Flyvbjerg (2006), case studies are valuable in the collective processes of knowledge generation and dissemination.

Considering these limitations, future research could build on our findings. 1) Replication/proof of concept is recommended using different (international) samples, educational programmes (e.g., applied university/vocational training) and other contexts. This is needed as our findings might be unique for the investigated sample/timing of data collection (during the COVID-19 pandemic). (2) Causal relationships and developments over time could be studied using genetic and/or longitudinal methods as this would increase the knowledge of possible predictive effects of health behavioural patterns on mental well-being and would add information for the development of interventions/services. 3) Earlier studies showed that individual health behaviours greatly impact biological mechanisms (e.g., metabolic and brain-based regulatory processes) involved in the development of mental well-being symptoms (Silverman and Deuster, 2014). Follow-up research could study how different health behavioural patterns influence biological mechanisms. 4) Interventions often focus on one health-risk behaviour. However, the clustering of health behaviours should be considered when developing new interventions, resulting in integrative human-centered approaches.

## 5 Conclusion

This study identified four health behavioural classes among Dutch university students and showed that the classes differed in socio-demographics and mental well-being. These insights shed new light on health behavioural patterns and associated factors and can be used to improve student support services and prevention/intervention measures.

## Availability of data and material

The data used in this manuscript are part of a larger ongoing longitudinal project (the Healthy Student Life project) and therefore not publicly available. Data are available on request.

## Funding

This work was supported by Radboud University.

## Ethical approval

This study was performed in line with the principles of the Declaration of Helsinki. Approval was granted by the Ethics Committee Social Sciences of Radboud University (ECSW-2021-086).

## Declaration of Competing Interest

The authors declare that they have no known competing financial interests or personal relationships that could have appeared to influence the work reported in this paper.

## Data Availability

Data will be made available on request.

## Footnotes

7 In this study, the general term “health behaviour” is used when referring to patterns, actions or habits affecting the health status of individuals (Conner and Norman, 2017). The term “health-risk behaviour” is used when specifically referring to actions linked to the increased likelihood of morbidity and mortality while the term “health-protective behaviour” is used when specifically referring to actions linked to reduced disease susceptibility or restoration of health (Spring et al., 2012).

8 Radboud University is a general research university that offers the highest level of tertiary education with an academic orientation. Different research-oriented bachelor’s and master’s programmes are offered in interdisciplinary fields. The university consists of 7 different faculties and in total enrolls over 24,000 students of which ±11% are international students (Radboud University, n.d.). There is no specific profile of university recruitment. The Dutch government offers financial support to all Dutch students and most students (those with a Dutch nationality or another nationality from a EU/EEA country, Switzerland or Suriname) pay a statutory tuition fee (determined by the Dutch government and the same for most Dutch universities). Consequently, there are only small differences in the quality of Dutch universities and education is available for everyone despite their socio-economic status. On average, the duration of a bachelor’s programme is 3 years and the duration of master’s programmes varies between 1 and 3 years. Additional information on the Dutch educational system and admission requirements can be consulted in the article by Luijkx and de Heus (2008).
