# Supplementary material for: Prevalence and clustering of health behaviours and the association with socio-demographics and mental well-being in Dutch university students
Source: Prev Med Rep. 2023 Jul 4;35:102307. doi: 10.1016/j.pmedr.2023.102307 (PMC10382923; doi:10.1016/j.pmedr.2023.102307)
Supplement: Supplementary data 1 [file mmc1.docx]

**Appendix A - Prevalence** **and Clustering of Health Behaviours and the Association with Socio-Demographics and Mental Well-being in Dutch University Students**

Journal: Preventive Medicine Reports

Author names: Kirsten J.M. van Hooijdonk^1^, Sterre S.H. Simons^1*^, Tirza H.J. van Noorden^1*^, Sabine A.E. Geurts^1^, Jacqueline M. Vink^1
1^Behavioural Science Institute, Radboud University, Nijmegen, the Netherlands
*Authors have contributed equally.
Correspondence: [kirsten.vanhooijdonk@ru.nl](mailto:kirsten.vanhooijdonk@ru.nl) (Kirsten J.M. van Hooijdonk)

**A.1. Measures**

***Health Behaviours***

*Physical activity* was assessed by using the International Physical Activity Questionnaire – Short Form (IPAQ-SF) items on the three activity categories: vigorous physical activity (e.g., activities that take high physical effort, make you sweat, and breathe much faster than normal), moderate physical activity (e.g., activities causing slightly increased sweating, and breathing) and walking (Craig et al., 2017; IPAQ Research Committee, 2005). Participants were asked to indicate during how many days of the past week they performed each activity. Additionally, the average number of hours and minutes per activity on such a day was asked, from which Metabolic Equivalent of Task (MET)-minutes/week scores were calculated per activity category. These MET-minutes/week scores were derived by multiplying the MET value per activity (8.0 for vigorous physical activity, 4.0 for moderate physical activity, 3.3 for walking) by the total duration of that activity per day in minutes and the amount of days per week that activity was performed (IPAQ Research Committee, 2005). Additionally, a total MET-minutes/week score was calculated by summing the MET-minutes/week scores of all three categories. Next, the MET-minutes/week scores of all three activity categories and days per week a certain activity was performed were used to categorize participants’ level of physical activity as (1) low, (2) moderate and (3) high, see the scoring protocol (IPAQ Research Committee, 2005). To summarize, participants were classified as (1) low when participants did not meet the criteria for categories (2) moderate or (3) high. Participants were classified as (2) moderate when (a) they had a vigorous-intensity activity of at least 20 minutes per day for ≥3 days per week, (b) they had a moderate-intensity activity and/or walking of at least 30 minutes per day for ≥5 days per week or (c) they had a minimal total MET-minutes/week score of 600 and performed any combination of vigorous physical activity/moderate physical activity/walking for ≥5 days per week. Participants were classified as (3) high when (a) they were vigorous physically active on ≥3 day per week achieving a minimal total MET-minutes/week score of 1500 or (b) they performed any combination of vigorous physical activity/moderate physical activity/walking for ≥7 days per week achieving a minimal total MET-minutes/week score of 3000. Last, data of participants whose total physical activity (all three categories combined) exceeded 16 hours a day were set to missing (n=23) (IPAQ Research Committee, 2005).

*Daily sitting time* was assessed by using the IPAQ-SF items on sitting (Craig et al., 2017; IPAQ Research Committee, 2005). Participants were asked to indicate how many hours and minutes per day they were sitting in the past 7 days. Based on this, their total sitting time per day was calculated. Participants who reported sitting more than 16 hours a day were set to missing (n=21) (IPAQ Research Committee, 2005). Participants’ daily sitting time was categorized into (1) 0 to <4 hours, (2) 4 to <6 hours, (3) 6 to <8 hours and (4) >8 hours, in line with former studies (Ekelund et al., 2019; Ekelund et al., 2016; Stamatakis et al., 2019).

*Eating habits – sweet snacks / savoury snacks / fruit and vegetable intake* were assessed based on the procedure described by van den Broek et al. (2020). Students were asked how many days in a normal week they consumed (a) pastry/cake/cookies, (b) candy bars, (c) chocolate, (d) candy, (e) potato crips, (f) warm/fried snacks, (g) fruit, (h) salad and raw vegetables and (i) heated vegetables. The maximum number of days per week *sweet snacks* were consumed was calculated by taking the maximum of categories (a-d). Similarly, the maximum number of days per week *savoury snacks* and *fruit and vegetables* were consumed was calculated for categories (e-f) and categories (g-i), respectively. All variables (range 0-7 days) were added separately to the analyses.

*Licit substance use – hazardous alcohol use* was assessed by using the Alcohol Use Disorder Identification Test – Consumption (AUDIT-C) (Bush et al., 1998). The questions were slightly adapted for the current study as mentioned in the pre-registration (van Hooijdonk et al., 2022): “The AUDIT-C (Bush et al., 1998) was modified for use in this study. Instead of asking about alcohol consumption in the past year, we narrowed this to the past half year (to match the longitudinal nature of the Healthy Student Life study). Regarding the question “How often did you have a drink containing alcohol in the past year?”, the “Never” category was divided into (1) Never and (2) Have been drinking, but not in the past half year. Regarding the question, “How often did you have 6 or more drinks on one occasion in the past year?” the “Never” category was divided into (1) “Never” and (2) “Have been drinking, but not in the past half year”, the “Less than monthly” category was included as (3) “Monthly or less”, the “Monthly” category was included as (4) “2 to 4 times a month”, the “Weekly” category was included as (5) “4 to 5 times a week” and the “Daily or almost daily” category was included as “6 to 7 times per week” in line with the answer categories of the first AUDIT-C question. Additionally, in the Netherlands binge drinking has been defined as 4 glasses per occasion for females and 6 glasses per occasion for males (Trimbos Instituut, 2022). Therefore, the amount of glasses per occasion has been adapted to 4 for females, and 5 for gender other (averaging the amount of glasses for males and females)”. For all participants, an AUDIT-C score was calculated based on the scoring protocol (Bush et al., 1998). To summarize, for questions “How often did you have a drink containing alcohol in the past year?” and “How often did you have 4/5/6 or more drinks on one occasion in the past half year?” participants received 0 points for answer categories (1) “Never” and (2) “Have been drinking in the past, but not in the past half year”, 1 point for answer category (3) “Monthly or less”, 2 points for answer category (4) “2 to 4 times a month”, 3 points for answer category (5) “2 to 3 times a week” and 4 points for answer categories (6) “4 to 5 times a week” and (7) “6 to 7 times per week”. For question “How many drinks did you have on a typical day when you were drinking in the past half year?” participants received 0 points if they answered 0-2 glasses, 1 point if they answered 3-4 glasses, 2 points if they answered 5-6 glasses, 3 points if they answered 7-9 glasses and 4 points if they answered ≥10 glasses. Next, scores on all three questions were summed and students were categorized as (1) low-risk (total score <7) or (2) at-risk for hazardous alcohol use (total score ≥7) (Verhoog et al., 2020).

*Licit substance use – tobacco and nicotine / cannabis / hookah* were assessed by asking how often students used (a) tobacco (cigarettes/shag), (b) electronic cigarettes/e-cigarettes, (c) cannabis and (d) hookah during the past half year. Answers included (1) “Never”, (2) “Ever taken a puff of a cigarette a few times” (only asked for category (a) tobacco), (3) “Ever used/smoked (regularly), but not in the past half year”, (4) “Monthly or less”, (5) “2 to 4 times a month”, (6) “2 to 3 times a week”, (7) “4 to 5 times a week”, (8) “6 to 7 times per week”. Three licit substance use variables were constructed using items (a-b) for *tobacco and nicotine,* item (c) for *cannabis* and item (d) for *hookah.* Per licit substance use category, the item with the highest frequency of use determined if students were classified as (1) non-user (when (1) or (2) was the highest frequency of use), (2) former user (when (3) was the highest frequency of use) or (3) recent user (when (4) to (8) was the highest frequency of use).

*Illicit substance use – party stimulant drugs / other stimulant drugs / sedative drugs* were assessed by asking how often students used (a) ecstasy, (b) designer drugs, (c) amphetamines, (d) cocaine, (e) Ritalin and/or Concerta (not on doctor’s prescription), (f) ketamine, (g) GHB and (h) sleeping and/or sedative drugs (not on doctor’s prescription) during the past half year. Answers included (1) “Never”, (2) “Ever used/smoked (regularly), but not in the past half year”, (3) “Monthly or less”, (4) “2 to 4 times a month”, (5) “2 to 3 times a week”, (6) “4 to 5 times a week”, (7) “6 to 7 times per week”. Three illicit substance use variables were constructed using items (a-b) for *party stimulant drugs,* items (c-e) for *other stimulant drugs* and items (f-h) for *sedative drugs.* Per illicit substance use category, the item with the highest frequency of use determined if students were classified as (1) non-user (when (1) was the highest frequency of use), (2) former user (when (2) was the highest frequency of use) or (3) recent user (when (3) to (7) was the highest frequency of use).

*Problematic internet use* was assessed by using the 6-item Problematic Internet Use Questionnaire Short-Form (PIUQ-SF-6) (Demetrovics et al., 2016). Cronbach’s alpha was 0.78 in the current study. Participants were asked to indicate how much each statement characterized them (e.g., “How often do you spend time online when you’d rather sleep?”) on a 5-point scale, ranging from (1) “Never” to (5) “Always/almost always”. A sum score of all items was calculated (range 6-30). Based on this score, students were categorized as (1) no-risk (total score <15) or (2) at-risk of problematic internet use (total score ≥15) (Demetrovics et al., 2016).

*Sleep – duration / quality* were assessed by asking about their (average) sleep *duration* per night before a work/study day (answers categories (1) "4 hours or less”, (2) “5 hours”, (3) “6 hours”, (4) “7 hours”, (5) “8 hours”, (6) “9 hours” and (7) “10 hours or more”) and about their (average) *quality* of sleep during those nights on a 5-point scale, ranging from (1) “very poor” to (5) “very good”. Both variables were added separately to the analyses.

***Socio-Demographics***

*Gender* was assessed by asking students’ gender, answers included (1) Male, (2) Female or (3) Other.

*Age* (in years) at the moment of completing the survey was constructed by subtracting date of birth from date of survey completion.

*Nationality* as mentioned in the participants’ passports was requested from the university administrative systems. Based on the definition used by Statistics Netherlands (Statistics Netherlands, 2022), two categories were created (1) Western nationality (including countries in Europe (excl. Turkey), Northern America and Oceania, and Indonesia and Japan) and (2) Non-western nationality (including countries in Africa, South America, and Asia (excl. Indonesia and Japan), or Turkey).

*International student* was defined as having a non-Dutch nationality and a non-Dutch pre-education. Based on the information for the university administrative systems, students were classified as international student (0) No or (1) Yes.

*Programme level* (1) bachelor, (2) pre-master or (3) master was obtained from the university administrative systems.

*Living situation – Alone / Roommate(s) / Parent(s) / Partner / Child(ren) / Other family members* were assessed by asking students with whom they lived during most of an average study week. Students could mark multiple answers from the following categories: (a) *Alone*, with (b) *Roommate(s)*, (c) *Parent(s)*, (d) *Partner*, (e) *Child(ren)*, (f) *Other family member(s)*. If students marked a category they were classified as (1) “Yes” for that category, if they did not mark a category they were classified as (0) “No” for that category. As a result they could be classified as (1) “Yes” in more than one category, for example when living with both parents and other family members, or with both partner and child(ren). All 6 variables were added separately to the analyses.

*Relationship status* was assessed by asking students to describe their current relationship status. Answers included (1) “Single”, (2) “Married”, (3) “In a steady relationship and living together”, (4) “In a steady relationship and living apart”, (5) “Dating without a steady relationship”, (6) “In a non-monogamous relationship”, (7) “Divorced” and (8) “Other”. In the analyses answers (3-4) were combined into the category steady relationship and the answers (6-8) were combined into the category other (due to low prevalence).

*LGBTIQ+ community* was assessed by asking if students recognize themselves as part of the LGBTIQ+ community. Answers included (1) “Yes” and (0) “No”.

*Membership student association* was assessed by asking if students were a member of a study association or student association. A study association was defined as an association linked to a particular study program, field of study and/or faculty. A student association was defined as a group of students with different study backgrounds focused on social activities, including fraternities and sororities. Answers included (1) “Yes, I am a member of a study association”, (2) “Yes, I am a member of a student association”, (3) “Yes, I am a member of both a study association and student association”, (4) “No, I'm not a member of a study association or student association”. Answers (1) and (4) were combined into (0) No member of student association and answers (2-3) were combined into (1) Member of student association.

*Body Mass Index (BMI)* was assessed by asking students to indicate their current height (in cm) and weight (in kg). Afterwards, a BMI score was calculated when data on both measures was available by dividing weight by height*height. BMI scores <10 and >60 were not deemed viable and therefore BMI scores of those participants were set to missing (n=8).

*Financial difficulty* was assessed by asking if students had difficulty paying for things (1 item of the Watson Perceived Financial Strain scale) (Watson et al., 2015). Answers were given on a 5-point scale, ranging from (1) “Strongly disagree” to (4) “Strongly agree”.

***Mental Well-being***

*Life satisfaction* was assessed by using the single-item measure of Cheung and Lucas (2014), which asked on a 4-point scale (ranging from (1) “Very dissatisfied” to (4) “Very Satisfied”) how satisfied students were (in general) with their life.

*Happiness* was assessed by using the single-item happiness measure of Abdel-Khalek (2006), which asked to which extent students felt happy (in general). The answer scale was slightly adapted to a 10-point scale (ranging from (1) “Not happy at all” to (10) “Very happy”).

*Burnout* was assessed by using the 12-item Short Burnout Assessment Tool (BAT-12) (Schaufeli et al., 2019). Cronbach’s alpha was 0.88 in the current study. Students were asked to indicate for each item (e.g., “I feel mentally exhausted”) how often this applied to them on a 5-point scale, ranging from (1) “Never” to (5) “Always”. A mean score was calculated where higher scores represent more burnout complaints.

*Perceived stress* was assessed by using the 10-item Perceived Stress Scale (PSS-10) (Cohen et al., 1994). Cronbach’s alpha was 0.88 in the current study. Students were asked to indicate for each item (e.g., “How often have you been upset because of something that happened unexpectedly?”) to which extent the statement applied to them during the last month on a 5-point scale, ranging from (0) “Never” to (4) “Very often”. After recoding 4 positive framed items, a sum score was calculated (range 0-40) where higher scores represent more perceived stress.

*Depression* was assessed by using the 8-item Center for Epidemiologic Studies - Depression Scale (CES-D-8) (Van de Velde et al., 2009). Cronbach’s alpha was 0.85 in the current study. Students were asked to indicate for each of the items how much of the time during the past week they experienced a described feeling (e.g., “depressed”) on a 4-point scale, ranging from (0) “None or almost none of the time” to (3) “All or almost all of the time”. Positively framed items were recoded and a sum score was calculated (range 0-24) where higher scores represent more depressive symptoms.

*Anxiety* was assessed by using the 2-item Generalized Anxiety Disorder scale (GAD-2) (Donker et al., 2011). Cronbach’s alpha was 0.80 in the current study. Students were asked to indicate for each item if they were bothered by the problems reflected in the items during the last 2 weeks (e.g., “Feeling nervous, anxious or on edge”) on a 4-point scale ranging from (0) “Not at all”, (1) “Several days”, (2) “More than half the days” to (3) “Nearly every day”. A sum score was calculated (range 0-6) where higher scores represent more anxiety symptoms.

*Study engagement* was assessed by using the 9-item shortened student version of the Utrecht Work Engagement Scale (UWES-S-9) (Schaufeli & Bakker, 2004). Cronbach’s alpha was 0.91 in the current study. This scale consists of 9 statements (e.g., “When I study, I feel like I am bursting with energy”) for which students were asked to indicate how often each statement applied to them on a 7-point scale, ranging from (0) “Never” to (6) “Always - Every day”. A mean score was calculated where higher scores represent more study engagement.

*COVID-19 concerns regarding social life / future prospects job market* were assessed by asking if students were concerned about (a) the impact of the COVID-19 outbreak on their *social life* and (b) on their *future prospects* *on the job market*. Both items where asked on a 5-point scale ranging from (1) “Strongly disagree” to (5) “Strongly agree” and separately included in the analyses.

**References**

Abdel-Khalek, A. M. (2006). Measuring happiness with a single-item scale. *Soc Behav Pers*, *34*(2), 139-150. <https://doi.org/10.2224/sbp.2006.34.2.139>

Bush, K., Kivlahan, D. R., McDonell, M. B., Fihn, S. D., & Bradley, K. A. (1998). The AUDIT alcohol consumption questions (AUDIT-C): an effective brief screening test for problem drinking. Ambulatory Care Quality Improvement Project (ACQUIP). Alcohol Use Disorders Identification Test. *Arch Intern Med*, *158*(16), 1789-1795. <https://doi.org/10.1001/archinte.158.16.1789>

Cheung, F., & Lucas, R. E. (2014). Assessing the validity of single-item life satisfaction measures: results from three large samples. *Qual Life Res*, *23*(10), 2809-2818. <https://doi.org/10.1007/s11136-014-0726-4>

Cohen, S., Kamarck, T., & Mermelstein, R. (1994). "Perceived stress scale." Measuring stress: A guide for health and social scientists. *10*(2), 1-2. <https://www.northottawawellnessfoundation.org/wp-content/uploads/2018/04/PerceivedStressScale.pdf>

Craig, C., Marshall, A., Sjostrom, M., Bauman, A., Lee, P., Macfarlane, D., Lam, T., & Stewart, S. (2017). International physical activity questionnaire-short form. *J Am Coll Health*, *65*(7), 492-501.

Demetrovics, Z., Király, O., Koronczai, B., Griffiths, M. D., Nagygyörgy, K., Elekes, Z., Tamás, D., Kun, B., Kökönyei, G., & Urbán, R. J. P. O. (2016). Psychometric properties of the Problematic Internet Use Questionnaire Short-Form (PIUQ-SF-6) in a nationally representative sample of adolescents. *PLoS One*, *11*(8), e0159409. <https://doi.org/10.1371/journal.pone.0159409>

Donker, T., van Straten, A., Marks, I., & Cuijpers, P. (2011). Quick and easy self-rating of Generalized Anxiety Disorder: validity of the Dutch web-based GAD-7, GAD-2 and GAD-SI. *Psychiatry Res*, *188*(1), 58-64. <https://doi.org/10.1016/j.psychres.2011.01.016>

Ekelund, U., Brown, W. J., Steene-Johannessen, J., Fagerland, M. W., Owen, N., Powell, K. E., Bauman, A. E., & Lee, I.-M. (2019). Do the associations of sedentary behaviour with cardiovascular disease mortality and cancer mortality differ by physical activity level? A systematic review and harmonised meta-analysis of data from 850 060 participants. *Br J Sports Med*, *53*(14), 886-894. <https://doi.org/10.1136/bjsports-2017-098963>

Ekelund, U., Steene-Johannessen, J., Brown, W. J., Fagerland, M. W., Owen, N., Powell, K. E., Bauman, A., & Lee, I. M. (2016). Does physical activity attenuate, or even eliminate, the detrimental association of sitting time with mortality? A harmonised meta-analysis of data from more than 1 million men and women. *Lancet*, *388*(10051), 1302-1310. <https://doi.org/10.1016/S0140-6736(16)30370-1>

IPAQ Research Committee. (2005). Guidelines for data processing and analysis of the International Physical Activity Questionnaire (IPAQ)-short and long forms. <https://sites.google.com/site/theipaq/scoring-protocol>

Schaufeli, W., & Bakker, A. (2004). UWES: Utrecht Work Engagement Scale: preliminary manual [version 1.1, December 2004]. <https://www.wilmarschaufeli.nl/publications/Schaufeli/Test%20Manuals/Test_manual_UWES_English.pdf>

Schaufeli, W., De Witte, H., & Desart, S. (2019). Handleiding Burnout Assessment Tool (BAT) – Versie 2.0. KU Leuven, België: Intern rapport. <https://burnoutassessmenttool.be/wp-content/uploads/2020/08/Gebruikshandleiding-BAT-versie-2.0.pdf>

Stamatakis, E., Gale, J., Bauman, A., Ekelund, U., Hamer, M., & Ding, D. (2019). Sitting Time, Physical Activity, and Risk of Mortality in Adults. *J Am Coll Cardiol*, *73*(16), 2062-2072. <https://doi.org/10.1016/j.jacc.2019.02.031>

Statistics Netherlands. (2022). *Wat is het verschil tussen een westerse en niet-westerse allochtoon?* Retrieved 7-12-2022 from <https://www.cbs.nl/nl-nl/faq/specifiek/wat-is-het-verschil-tussen-een-westerse-en-niet-westerse-allochtoon-#:~:text=Westers%3A,Indonesi%C3%AB%20en%20Japan)%20of%20Turkije>

Trimbos Instituut. (2022). *Wat is binge drinken?* <https://www.alcoholinfo.nl/feiten/wat-is-binge-drinken#:~:text=Binge%20drinken%20wordt%20ook%20wel,iemand%20op%20losbandige%20manier%20consumeert>.

Van de Velde, S., Levecque, K., & Bracke, P. (2009). Measurement equivalence of the CES-D 8 in the general population in Belgium: a gender perspective. *Arch Public Health*, *67*(1), 14-29. <https://doi.org/10.1186/0778-7367-67-1-15>

van den Broek, N., Larsen, J. K., Verhagen, M., Burk, W. J., & Vink, J. M. (2020). Is Adolescents’ Food Intake Associated with Exposure to the Food Intake of Their Mothers and Best Friends? *Nutrients*, *12*(3), 786. <https://doi.org/10.3390/nu12030786>

van Hooijdonk, K. J. M., Vink, J. M., Geurts, S. A. E., van Noorden, T. H. J., & Simons, S. S. H. (2022). *Preregistration Healthy Student Life project - Clustering of health behaviours and mental health status in Dutch university students: Results of a Cross-Sectional study*. Open Science Framework. Retrieved 7-12-2022 from <https://doi.org/10.17605/OSF.IO/M48XZ>

Verhoog, S., Dopmeijer, J. M., de Jonge, J. M., van der Heijde, C. M., Vonk, P., Bovens, R. H. L. M., de Boer, M. R., Hoekstra, T., Kunst, A. E., Wiers, R. W., & Kuipers, M. A. G. (2020). The Use of the Alcohol Use Disorders Identification Test – Consumption as an Indicator of Hazardous Alcohol Use among University Students. *Eur Addict Res*, *26*(1), 1-9. <https://doi.org/10.1159/000503342>

Watson, S. J., Barber, B. L., & Dziurawiec, S. (2015). The role of economizing and financial strain in Australian university students’ psychological well-being. *J Fam Econ Iss*, *36*, 421-433. <https://doi.org/10.1007/s10834-014-9404-5>

**A.2. Descriptive Statistics Mental Well-being**

All mean well-being scores and information on norm scores (if available) have been presented in Table A.2. The observed mean scores on life satisfaction and happiness were above the scale average indicating that overall students were in general satisfied with their life and felt in general happy. Burnout symptoms were observed in the sample, as the mean observed score for burnout was above the scale average. Symptoms of perceived stress, depression and anxiety were also observed as the mean scores of these scales were approaching the scale average. Regarding study engagement, the mean observed score was slightly above the scale average, indicating that students felt moderately engaged with their studies. Last, the mean observed scores for COVID-19 concerns were above the scale average. This suggests that students were moderately concerned about the impact of the COVID-19 pandemic on their social life and contacts, and their future prospects on the job market.

**Table A.2.**

Descriptive Statistics Mental Well-being among Dutch university students (n=3771) part of Healthy Student Life project (survey Oct-Nov 2021).

|  | | **Total sample**  Mean (SD; n) | **Scale** | **Norm score (if available)*** |
| --- | --- | --- | --- | --- |
| Life satisfaction *(Range 1-4)* | | 3.0 (0.6; 3770) | Single-item Cheung and Lucas (2014) | N/A |
| Happiness *(Range 1-10)* | | 6.9 (1.5; 3770) | Adapted version single-item Abdel-Khalek (2006) | N/A |
| Burnout *(Mean;* *Range 1-5)* | | 2.7 (0.7; 3770) | 12-item Short Burnout Assessment Tool (BAT-12) (Schaufeli et al., 2019) | No burnout: <2.53  There is a risk of burning out: ≥2.54 to <2.96  Burnout is most likely: ≥2.96  (Schaufeli et al., 2019) |
| Perceived stress *(Sum;* *Range 0-40)* | | 16.9 (6.9; 3770) | 10-item Perceived Stress Scale (PSS-10) (Cohen et al., 1994) | Low self-perceived stress score: 0 to 13  Moderate self-perceived stress score: 14 to 26  High self-perceived stress score: 27 to 40  (Torales et al., 2020) |
| Depression *(Sum; Range 0-24)* | | 8.5 (4.5; 3771) | 8-item Center for Epidemiologic Studies - Depression Scale (CES-D-8) (Van de Velde et al., 2009) | Clinically significant depressive symptoms: ≥9  (Briggs et al., 2018) |
| Anxiety *(Sum; Range 0-6)* | | 2.3 (1.7; 3770) | 2-item Generalized Anxiety Disorder scale (GAD-2) (Donker et al., 2011) | Clinically significant anxiety symptoms: ≥3  (Sapra et al., 2020) |
| Study engagement *(Mean; Range 0-6)* | | 3.5 (1.1; 3771) | 9-item shortened student version of the Utrecht Work Engagement Scale (UWES-S-9) (Schaufeli & Bakker, 2004). | Very low engagement: ≤ 1.77  Low engagement: 1.78 to 2.88  Average engagement: 2.89 to 4.66  High engagement: 4.67 to 5.50  Very High engagement: ≥ 5.51  (Schaufeli & Bakker, 2004). |
| COVID-19 concerns |  |  |  |  |
| Social life and contacts *(Range 1-5)* | | 3.5 (1.2; 3626) | Self-constructed | N/A |
| My future prospects in the job market *(Range 1-5)* | | 2.8 (1.2; 3627) | Self-constructed | N/A |

Note. Not all available norm scores were constructed for the target population. Consequently, scores should be interpreted with caution.

**References**

Abdel-Khalek, A. M. (2006). Measuring happiness with a single-item scale. *Soc Behav Pers*, *34*(2), 139-150. https://doi.org/10.2224/sbp.2006.34.2.139

Briggs, R., Carey, D., O’Halloran, A. M., Kenny, R. A., & Kennelly, S. P. (2018). Validation of the 8-item Centre for Epidemiological Studies Depression Scale in a cohort of community-dwelling older people: data from The Irish Longitudinal Study on Ageing (TILDA). *European Geriatric Medicine*, *9*, 121-126. https://doi.org/10.1007/s41999-017-0016-0

Cheung, F., & Lucas, R. E. (2014). Assessing the validity of single-item life satisfaction measures: results from three large samples. *Qual Life Res*, *23*(10), 2809-2818. https://doi.org/10.1007/s11136-014-0726-4

Cohen, S., Kamarck, T., & Mermelstein, R. (1994). "Perceived stress scale." Measuring stress: A guide for health and social scientists. *10*(2), 1-2. https://www.northottawawellnessfoundation.org/wp-content/uploads/2018/04/PerceivedStressScale.pdf

Donker, T., van Straten, A., Marks, I., & Cuijpers, P. (2011). Quick and easy self-rating of Generalized Anxiety Disorder: validity of the Dutch web-based GAD-7, GAD-2 and GAD-SI. *Psychiatry Res*, *188*(1), 58-64. <https://doi.org/10.1016/j.psychres.2011.01.016>

Sapra, A., Bhandari, P., Sharma, S., Chanpura, T., & Lopp, L. (2020). Using generalized anxiety disorder-2 (GAD-2) and GAD-7 in a primary care setting. *Cureus*, *12*(5). https://doi.org/ [10.7759/cureus.8224](https://doi.org/10.7759%2Fcureus.8224)

Schaufeli, W., & Bakker, A. (2004). UWES: Utrecht Work Engagement Scale: preliminary manual [version 1.1, December 2004]. https://www.wilmarschaufeli.nl/publications/Schaufeli/Test%20Manuals/Test_manual_UWES_English.pdf

Schaufeli, W., De Witte, H., & Desart, S. (2019). Handleiding Burnout Assessment Tool (BAT) – Versie 2.0. KU Leuven, België: Intern rapport. https://burnoutassessmenttool.be/wp-content/uploads/2020/08/Gebruikshandleiding-BAT-versie-2.0.pdf

Torales, J., Ríos-González, C., Barrios, I., O'Higgins, M., González, I., García, O., ... & Ventriglio, A. (2020). Self-perceived stress during the quarantine of COVID-19 pandemic in Paraguay: an exploratory survey. *Frontiers in psychiatry*, *11*, 558691. https://doi.org/[10.3389/fpsyt.2020.558691](https://doi.org/10.3389%2Ffpsyt.2020.558691)

Van de Velde, S., Levecque, K., & Bracke, P. (2009). Measurement equivalence of the CES-D 8 in the general population in Belgium: a gender perspective. *Arch Public Health*, *67*(1), 14-29. https://doi.org/10.1186/0778-7367-67-1-15

**A.3. Probabilities latent class analyses**

**Table A.3.**

Probabilities latent class analyses among Dutch university students (n=3771; subsample of Healthy Student Life survey Oct-Nov 2021

| **Health behaviour** |  | Class 1 | Class 2 | Class 3 | Class 4 |
| --- | --- | --- | --- | --- | --- |
| Physical activity | Low | 0.0948 | 0.0660 | 0.0903 | 0.1454 |
|  | Moderate | 0.3895 | 0.3791 | 0.4569 | 0.4430 |
|  | High | 0.5157 | 0.5549 | 0.4528 | 0.4116 |
| Daily sitting time | 0 to <4 hours | 0.0466 | 0.0315 | 0.0216 | 0.0425 |
|  | 4 to <6 hours | 0.1352 | 0.1412 | 0.0950 | 0.1135 |
|  | 6 to <8 hours | 0.2124 | 0.2465 | 0.2341 | 0.2224 |
|  | ≥8 hours | 0.6058 | 0.5808 | 0.6493 | 0.6215 |
| Eating habits |  |  |  |  |  |
| Sweet snacks | 0 days | 0.0684 | 0.1049 | 0.0543 | 0.0732 |
|  | 1 day | 0.3164 | 0.3790 | 0.2383 | 0.1783 |
|  | 2 days | 0.2711 | 0.2436 | 0.2452 | 0.2346 |
|  | 3 days | 0.1495 | 0.1557 | 0.1895 | 0.2008 |
|  | 4 days | 0.0655 | 0.0451 | 0.0919 | 0.1206 |
|  | 5 days | 0.0720 | 0.0409 | 0.0855 | 0.0891 |
|  | 6 days | 0.0357 | 0.0000 | 0.0386 | 0.0403 |
|  | 7 days | 0.0214 | 0.0308 | 0.0568 | 0.0631 |
| Savoury snacks | 0 days | 0.0728 | 0.0721 | 0.0927 | 0.0797 |
|  | 1 day | 0.3661 | 0.3172 | 0.3809 | 0.2991 |
|  | 2 days | 0.3020 | 0.3005 | 0.3071 | 0.2946 |
|  | 3 days | 0.1492 | 0.1594 | 0.1402 | 0.1584 |
|  | 4 days | 0.0655 | 0.1011 | 0.0534 | 0.0943 |
|  | 5 days | 0.0353 | 0.0360 | 0.0125 | 0.0569 |
|  | 6 days | 0.0017 | 0.0114 | 0.0088 | 0.0104 |
|  | 7 days | 0.0074 | 0.0023 | 0.0045 | 0.0067 |
| Fruit and vegetables | 0 days | 0.0050 | 0.0065 | 0.0007 | 0.0051 |
|  | 1 day | 0.0074 | 0.0025 | 0.0014 | 0.0088 |
|  | 2 days | 0.0204 | 0.0083 | 0.0051 | 0.0319 |
|  | 3 days | 0.0388 | 0.0322 | 0.0288 | 0.0652 |
|  | 4 days | 0.0904 | 0.0775 | 0.0555 | 0.1222 |
|  | 5 days | 0.1306 | 0.1504 | 0.1177 | 0.1973 |
|  | 6 days | 0.2229 | 0.2622 | 0.1870 | 0.1909 |
|  | 7 days | 0.4844 | 0.4604 | 0.6037 | 0.3784 |
| Licit substance use |  |  |  |  |  |
| Hazardous alcohol use | Low-risk | 0.8475 | 0.7280 | 0.9771 | 0.9636 |
|  | At-risk | 0.1525 | 0.2720 | 0.0229 | 0.0364 |
| Tobacco/nicotine | Non-user | 0.5110 | 0.2808 | 0.9894 | 0.9009 |
|  | Former user | 0.1272 | 0.1235 | 0.0023 | 0.0128 |
|  | Recent user | 0.3618 | 0.5958 | 0.0082 | 0.0863 |
| Cannabis | Non-user | 0.0947 | 0.0853 | 0.8397 | 0.7879 |
|  | Former user | 0.4084 | 0.2968 | 0.0952 | 0.0986 |
|  | Recent user | 0.4970 | 0.6178 | 0.0651 | 0.1135 |
| Hookah | Non-user | 0.4689 | 0.4878 | 0.9627 | 0.9571 |
|  | Former user | 0.4216 | 0.4276 | 0.0294 | 0.0285 |
|  | Recent user | 0.1095 | 0.0847 | 0.0080 | 0.0143 |
| Illicit substance use |  |  |  |  |  |
| Party stimulant drugs | Non-user | 0.7060 | 0.0363 | 0.9657 | 0.9890 |
|  | Former user | 0.1786 | 0.2935 | 0.0172 | 0.0110 |
|  | Recent user | 0.1154 | 0.6703 | 0.0171 | 0.0000 |
| Other stimulant drugs | Non-user | 0.9195 | 0.1612 | 0.9857 | 0.9922 |
|  | Former user | 0.0739 | 0.4016 | 0.0128 | 0.0062 |
|  | Recent user | 0.0066 | 0.4372 | 0.0014 | 0.0016 |
| Sedative drugs | Non-user | 0.9137 | 0.3456 | 0.9722 | 0.9268 |
|  | Former user | 0.0486 | 0.3700 | 0.0151 | 0.0269 |
|  | Recent user | 0.0377 | 0.2844 | 0.0127 | 0.0463 |
| Problematic internet use | No-risk | 0.6837 | 0.7001 | 0.7933 | 0.5430 |
|  | At-risk | 0.3163 | 0.2999 | 0.2067 | 0.4570 |
| Sleep |  |  |  |  |  |
| Duration | ≤4h | 0.0082 | 0.0000 | 0.0000 | 0.0346 |
|  | 5h | 0.0288 | 0.0488 | 0.0031 | 0.1161 |
|  | 6h | 0.1449 | 0.1680 | 0.0498 | 0.3771 |
|  | 7h | 0.4156 | 0.4163 | 0.3647 | 0.3759 |
|  | 8h | 0.3475 | 0.2920 | 0.4860 | 0.0591 |
|  | 9h | 0.0386 | 0.0686 | 0.0932 | 0.0221 |
|  | ≥10h | 0.0165 | 0.0063 | 0.0031 | 0.0151 |
| Quality | Very poor | 0.0401 | 0.0197 | 0.0000 | 0.0859 |
|  | Poor | 0.1520 | 0.1684 | 0.0386 | 0.3321 |
|  | Not really good but not bad either | 0.3555 | 0.3680 | 0.2686 | 0.4612 |
|  | Pretty good | 0.3803 | 0.3967 | 0.5689 | 0.1019 |
|  | Very good | 0.0720 | 0.0472 | 0.1239 | 0.0189 |

Note. Probabilities per health behaviour and per response category are presented for all 15 health behaviours included in the Latent Class Analysis. Class 1 (n=862): “Illicit and licit substance use health-risk group”. Class 2 (n=435): “Hazardous alcohol use, (il)licit substance use, physically active”. Class 3 (n=1876): “Health-protective group”. Class 4 (n=598): “Non-substance use health-risk group”.
